# Supplementary material for: Novel Driver Strength Index highlights important cancer genes in TCGA PanCanAtlas patients
Source: PeerJ. 2022 Aug 11;10:e13860. doi: 10.7717/peerj.13860 (PMC9375969; doi:10.7717/peerj.13860)
Supplement: Supplemental Information 2 [file peerj-10-13860-s002.zip › COHORTS/genes plots/detailed/2021_8_16_14_9_distribution_events_detailed_males_36.pdf]

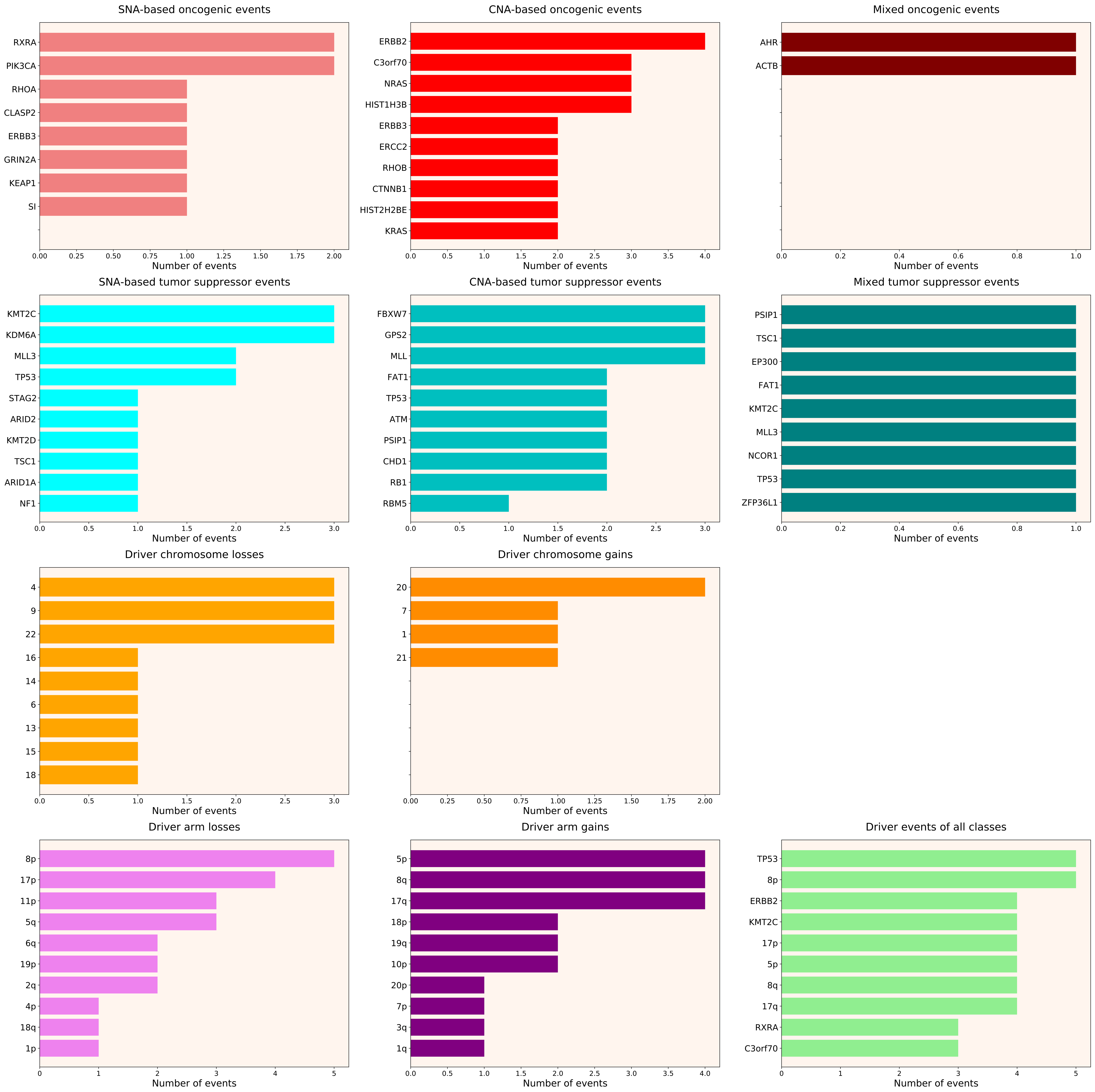
SNA-based oncogenic events
RXRA
PIK3CA
RHOA
CLASP2
ERBB3
GRIN2A
KEAP1
SI
0.00 0.25 0.50 0.75 1.00 1.25 1.50 1.75 2.00
Number of events
CNA-based oncogenic events
ERBB2
C3orf70
NRAS
HIST1H3B
ERBB3
ERCC2
RHOB
CTNNB1
HIST2H2BE
KRAS
0.0 0.5 1.0 1.5 2.0 2.5 3.0 3.5 4.0
Number of events
Mixed oncogenic events
AHR
ACTB
0.0 0.2 0.4 0.6 0.8 1.0
Number of events
SNA-based tumor suppressor events
KMT2C
KDM6A
MLL3
TP53
STAG2
ARID2
KMT2D
TSC1
ARID1A
NF1
0.0 0.5 1.0 1.5 2.0 2.5 3.0
Number of events
CNA-based tumor suppressor events
FBXW7
GPS2
MLL
FAT1
TP53
ATM
PSIP1
CHD1
RB1
RBM5
0.0 0.5 1.0 1.5 2.0 2.5 3.0
Number of events
Mixed tumor suppressor events
PSIP1
TSC1
EP300
FAT1
KMT2C
MLL3
NCOR1
TP53
ZFP36L1
0.0 0.2 0.4 0.6 0.8 1.0
Number of events
Driver chromosome losses
4
9
22
16
14
6
13
15
18
0.0 0.5 1.0 1.5 2.0 2.5 3.0
Number of events
Driver chromosome gains
20
7
1
21
0.00 0.25 0.50 0.75 1.00 1.25 1.50 1.75 2.00
Number of events
Driver arm losses
8p
17p
11p
5q
6q
19p
2q
4p
18q
1p
0 1 2 3 4 5
Number of events
Driver arm gains
5p
8q
17q
18p
19q
10p
20p
7p
3q
1q
0.0 0.5 1.0 1.5 2.0 2.5 3.0 3.5 4.0
Number of events
Driver events of all classes
TP53
8p
ERBB2
KMT2C
17p
5p
8q
17q
RXRA
C3orf70
0 1 2 3 4 5
Number of events
